# Supplementary material for: Humans peeing: Justice-involved women’s access to toilets in public spaces
Source: PLoS One. 2023 Mar 10;18(3):e0282917. doi: 10.1371/journal.pone.0282917 (PMC10004595; doi:10.1371/journal.pone.0282917)
Supplement: Attachment — Submitted filename: PLOS One Review 12-24-22.pdf [file pone.0282917.s001.pdf]

December 23, 2022

Re: Research Article Review

Dear Dr. Ismail

I want to thank you for extending the invitation to review the manuscript titled, "I am a human being: Justice-involved women's access to toilets in public spaces" for publication in the PLOS ONE journal. I have taken the time to extensively read and review the article and below are comments that I would like the authors of this manuscript to consider addressing. I truly enjoyed reading this paper and I support and recommend this article for publication with minor revisions (see attached comments).

Overall, I believe this paper provides a great overview of the limited access and discriminatory practices that revolved around the politics of toilet access. It raises really important issues on surveillance of toilet behaviors, gatekeepers that dictate access to toilets (highlighting the social power that gatekeepers have becoming a barrier to basic sanitation access), the productivity demands, environment of surveillance (when and who accesses a toilet). This paper makes the following conceptual contributions: Addresses an underexplored population that remains invisible and underserved, that is justice-involved women. It highlights vulnerable identities of justice-involved women, with women who engage in sex work, who have prior history of homelessness, and elderly women who are faced with challenges in in access a basic human right: access to sanitation. Lastly, the article provides powerful evidence to suggest that access to a toilet reinforces a cycle of poverty, criminalization, and homelessness, as inadequate access to toilets inhibits women's ability to safely navigate public places which may, in turn, limit their ability to meet the requirements of community supervision and reentry, including attending social services and treatment appointments, and acquiring and maintaining employment. It also increases the risk of women to practice public urination and defecation that can result in citation and a record of public indecency, which can potentially lead to a parole violation. The paper highlights the politics of a toilet outside the private sphere of the home and challenges us to think more critically about the importance of expanding public services to not only serve vulnerable populations that do not have basic access to water, sanitation, and hygiene, but that the general population could also greatly benefit.

Thank you for your time and consideration.

Sincerely yours,

Reviewer 1

Reviewer 1	**Major comments to consider:**
R1C1	Line 30: Bathroom(s) is a word used in the US only. If targeting a broader audience, the paper needs to define the words: bathroom, restroom, and toilet. Throughout the paper, authors interchangeably use "toilets," "restroom," and "bathroom" which they do not mean the same thing. I would recommend authors to first, be consistent with the words used throughout the paper. If the focus is on toilet access, then define it based on the WHO/UNICEF Joint Monitoring Programme (JMP) definition of securing basic access to sanitation. But do not go back and forth without really defining what a bathroom or restroom mean throughout the paper as these words do not mean the same thing and are not used worldwide. For WaSH scholars, the word used is typically sanitation, which refers to toilets. Would recommend doing more reading on the definitions to include in the paper. There is some literature that states bathroom refers to the private home vs. restroom refers to public facilities with a toilet and a sink to practice hand hygiene. It is important to distinguish and clearly define each of these terms. https://washdata.org/monitoring.<br>Line 119: remove bathroom, it should be toilet access. Be consistent and revise throughout the paper based on definitions.
R1C2	Lines 169-180: Consider moving to the results section. It does not belong in the methods section as this is a finding (pop demographics). Similar with lines 230-236, starting with "All the bathroom were located…" And ending with "private institutions (n- 11, 55%)," could be moved to the results when referring to audit of toilets. This is a result and does not belong in methods section either.
R1C3	Line 178: Define "ever being homeless." Does this mean that if at any time in their lives these women have experienced homelessness? In the past 6-months? Or are they currently experiencing homelessness?
R1C4	Line 178-179: Not clear from this pop characteristics, the number of women who are unstably housed or currently experiencing homelessness and need to specify in the results section. It would also be helpful to state the number of women who are housed (either living with family, with friends), shelter systems, and or who were unhoused at the time study activities were conducted. It would also be helpful to include who is employed and unemployed (aside from describing the work experiences), to provide a bit more information on population characteristics to understand the population without identifying personal information about these study participants. This would also help contextualize women's different access to sanitation based on place of employment described in section located in 318.
R1C5	Lines 287-291: Highlights an important argument and would like to see if the authors could expand on this point about discriminatory practices. What about the Black, Latina/Latine, and other underrepresented racial/ethnic women in the sample, did they highlighted any experience any of discrimination when trying to access a toilet in public spaces? Incorporate an intersectional analysis. What about women in different age groups, which this is slightly highlighted in lines 497 - 505. Highlight what are the intersectional vulnerable identities among these justice-involved women?
R1C6	Are there any women who menstruate in the sample population and do these women encounter challenges in menstrual hygiene management with limited access to a toilet? This is helpful to know given that on line 552 there is a mention on audit of toilets that rarely accommodate people who menstruate.
R1C7	Line 640: "Quality of life" charges, do you mean sleeping outdoors? Loitering? Would be helpful to define and highlight what city ordinances exist in the town where this study takes place and that perpetuates a cycle of poverty.
R1C8	Line 545: Recommend authors to highlight what is consider an excellent (point 5) facility. What do these facilities/toilets need to have been ranked a 1 vs 5-point? Helpful to describe in the methods section. Authors mentioned this is adapted from Maroko et al. (2021) article but should consider including a description of this point classification in the methods.

R1C9	Line 518: Is there publicly data available on the total number of public toilet facilities available in the town where this study takes place? To understand how many were not audited/missed. Authors could consider looking into this but not necessary, to highlight that only a portion of facilities were audited and not all. I do appreciate the authors including in Line 531self-reflexivity given authors' different status and positionality vs. that of study participants which may result in different experiences of access to a toilet in public spaces and places.
R1C10	Line 654: Should be "sanitation services" consider removing hygiene services if only focusing on toilet access. This is based on JMP definitions. Authors need to be consistent with the way bathroom, restroom, and toilet are being utilized throughout the paper because they do not mean the same thing. If referring to both sanitation and menstrual hygiene, then consider including both.
R1C11	Consider reading "No-Place to Go" audit report on toilet access in Skid Row, Los Angeles, CA and the Dirty Divide: Out of Service report by LACAN in Skid Row, Los Angeles, CA to connect existing studies in the discussion with the study contributions. At the same, consider engaging debates about urban space, homelessness, criminalization, and public service provision by including the work of Eugene McCann, Jessie Speer, Erin Goodling, Jennifer Wolsch, Don Mitchell, Forrest Stuart, Kelly Lytle Hernandez, Alheli Calderon-Villarreal, Lourdes Johanna Avelar Portillo, Shawn Flanigan, and Megan Welsh. The work of these authors provides helpful insights and discussions that are absent in this manuscript and can make the discussion stronger.
	**Minor comments to consider:**
R1C12	Line 40: I would clearly define the word "justice involved women" could write, justice involved women refers to women who…as not everyone may know the term and helpful for broader audience.
R1C13	I think it would be important to contextualize the location of this small town. If the issue of not digging deeper is to maintain confidentiality, then I would understand, but it is always important to understand the location of where this study is based on. Is this a rural town, how many people in this town and how many of those are unhoused/people experiencing homelessness? Important to know what the number of justice-involved women in the town is particularly when trying to fill knowledge gaps. This should go somewhere in the background section before line 81. Authors briefly mention this in line 584 but it takes many pages to learn a bit about the town.
R1C14	Line 59-60: Authors mention that "Black and Latina women are disproportionately incarcerated, and the majority of incarcerated women are white." Based on your study findings, it would be important to highlight this in the results section. Are there any differences in access and experiences of discrimination among the women who participated in the focus groups in their access to public/private toilets? This would make the analysis stronger. Incorporating an **intersectionality** analytical lens could also help make the results and discussion stronger (see major comment R1C5).
R1C15	Line 121-131: Could highlight better literature on WaSH insecurity, particularly literature on psychosocial distress related to limited access to sanitation and hygiene services. There are existing studies that explore the intersections of sanitation and hygiene insecurity and psychosocial stress among women.
R1C16	Consider citing article on Kuhlmann et al. (2019) on menstrual hygiene for low-income women in the US that may be useful to include in both literature/background section and discussion.
R1C17	Line 30: remove "to" in the sentence "limited to access" as it should read limited access to ____.

R1C18	Lines 121, 257: Consider indenting paragraphs in each of these lines to match style throughout the paper.
R1C19	Line 93-95: Include sources for those arguments made about racial segregation in public toilets and American Disability Act.
R1C20	Line 176: Please expand: engaged in sex work in the past or currently sex workers (meaning that at the time during research activities, they were sex workers)?
R1C21	Line 179: I would suggest defining "experiencing sleeping outside." Meaning sleeping in tents, vehicles, makeshifts, or abandoned buildings? Recommend looking into the HUD's definition of homelessness.
R1C22	Line 267: I would suggest defining what do you mean by geography and how is it different from location, meaning it is a small city, topography, etc.
R1C23	Line 515: "Policies that constructed voiding as optional" do authors mean "as an option" since there is a level of distrust that limits access to a toilet? Would help to expand this point a bit.
R1C24	Throughout the paper, in lines 234, 549, 561, authors use the term, "gender-free" but do they mean "gender neutral toilets"? Consider changing.
