# Supplementary material for: Humans peeing: Justice-involved women’s access to toilets in public spaces
Source: PLoS One. 2023 Mar 10;18(3):e0282917. doi: 10.1371/journal.pone.0282917 (PMC10004595; doi:10.1371/journal.pone.0282917)
Supplement: Attachment — Submitted filename: Response to Reviewers_PONE-S-22-38235_021923.pdf [file pone.0282917.s002.pdf]

r	Comment	Response
Editor	Please ensure that your manuscript meets PLOS ONE's style requirements, including those for file naming.	Figure Label = Fig 1; Table 1
Editor	In the ethics statement in the Methods, you have specified that verbal consent was obtained. Please provide additional details regarding how this consent was documented and witnessed, and state whether this was approved by the IRB	Additional detail about consent process was added to Methods section, page 8-9, lines 175 - 186. On page 8, Line 166-167, we have already included statement explaining that protocol was reviewed and approved by IRB. No additional language has been added.
Editor	Please expand the acronym "NIDDK/NIH" (as indicated in your financial disclosure) so that it states the name of your funders in full.	National Institute of Diabetes and Digestive and Kidney Diseases/National Institute of Health
Editor	Please review your reference list to ensure that it is complete and correct. If you have cited papers that have been retracted, please include the rationale for doing so in the manuscript text, or remove these references and replace them with relevant current references. Any changes to the reference list should be mentioned in the rebuttal letter that accompanies your revised manuscript. If you need to cite a retracted article, indicate the article's retracted status in the References list and also include a citation and full reference for the retraction notice.	The reference list is complete and correct. No papers have been retracted. Several references have been added, per recommendation of reviewers.
Editor	We note you have included a table to which you do not refer in the text of your manuscript. Please ensure that you refer to Table 1 in your text; if accepted, production will need this reference to link the reader to the Table	Reference to Table 1 has been added to the Toilet Audit Findings section: pg 25, line 707; pg 26, line 729; pg 27, line 759.
Reviewer #3	The statement on author contribution states 'Each author contributed in different ways to this work'. – This statement is not detailed enough. Please check submission guidelines.	Authors' roles were added to pg 1, lines 15-21.
Comments about data availability		
	In your Data Availability statement, you have not specified where the minimal data set underlying the results described in your manuscript can be found. PLOS defines a study's minimal data set as the underlying data used to reach the conclusions drawn in the manuscript and any additional data required to replicate the reported study findings in their entirety. All PLOS journals require that the minimal data set be made fully available. For more information about our data policy, please see http://journals.plos.org/plosone/s/data-availability.	

Editor	Upon re-submitting your revised manuscript, please upload your study's minimal underlying data set as either Supporting Information files or to a stable, public repository and include the relevant URLs, DOIs, or accession numbers within your revised cover letter. For a list of acceptable repositories, please see http://journals.plos.org/plosone/s/data-availability#loc-recommended-repositories. Any potentially identifying patient information must be fully anonymized.	
	Important: If there are ethical or legal restrictions to sharing your data publicly, please explain these restrictions in detail. Please see our guidelines for more information on what we consider unacceptable restrictions to publicly sharing data: http://journals.plos.org/plosone/s/data-availability#loc-unacceptable-data-access-restrictions. Note that it is not acceptable for the authors to be the sole named individuals responsible for ensuring data access.	The qualitative interview data are not publicly available due to privacy and safety concerns for the population. Publically sharing these transcripts would be a violation of the agreement to which the participants consented. Excerpts of the transcripts relevant to the study have been made available within the paper. The Southern Connecticut State Univeristy Institutional Review Board may be contacted for more information and data requests. (Contact: IRB@southernct.edu)
	We will update your Data Availability statement to reflect the information you provide in your cover letter.	
	We note that you have stated that you will provide repository information for your data at acceptance. Should your manuscript be accepted for publication, we will hold it until you provide the relevant accession numbers or DOIs necessary to access your data. If you wish to make changes to your Data Availability statement, please describe these changes in your cover letter and we will update your Data Availability statement to reflect the information you provide.	This study does not have a "minimal data set" that can be made more fully available. Yes, we want to make changes to our Data Availability statement. These changes are describe in our cover letter.
Reviewer #1	My only concern was that the data has some restrictions, yet the information on how to receive the unrestricted data had not been provided. However, I believe now that the data that is available is actually contained within the manuscript (toilet audit), and the data that is restricted is all of the focus group findings. Therefore the available data has already been shared, satisfying the criteria.	This interpretation is correct.

Reviewer #3	Data availability statement is not specific enough. States – 'No - some restrictions will apply' – but it is not made clear what the restrictions are. It appears that they are not making the data available at all, so this needs clarification and matching up with the following statement given by the authors 'Qualitative focus group data cannot be shared publicly because of privacy and safety concerns for the population'. In general FGD data can be shared as usually anonymous…(I agree these particular participants may have mitigating circumstances so it would be useful to amend this statement if need be. But as no names were taken and pseudonyms used then it is not made clear why the data would compromise privacy or safety. There seems to be little data in the paper that might identify any individual? Indeed, in the methods it also states During the transcription process, any identifying information (i.e., names, locations, dates) were deleted to create a de-identified data set.	
Reviewer #2	It seems like the broad framing of much of the intro and literature review does not match the very narrow focus of the survey instrument on the bladder. However, the responses from the participants seem to again broaden the scope. Could you give some examples of the types of questions in this interview instrument and talk about how the focus groups broadened the conversation?	Information about the interview instrument and examples of questions are already included on page 9, lines 250-275. An additional sentence was added here to expand this description.
Reviewer #2	In your literature review, you might want to frame your study as occurring in the context of decreasing access to bathrooms in most cities in the United States.	A sentence about the decreasing access to bathrooms was added to the introduction (p. 5, lines 125-127)
Reviewer #2	Did your focus groups include any transgender individuals? Could you elaborate on any of their experiences or what additional barriers they might face?	In the demographics survey, we did not ask participants if they identified as transgender or had a transgender experience. This is a limitation that we have added to the Limitation section (pg. 27-28). None of the participants talked about their gender identity as being a barrier to toilet access, but we did not specifically solicit this information.
Reviewer #2	The use of "void/voiding" was awkward to me, and it honestly took me a few pages to understand the meaning of the word as you are using it. I think "urination" and "defecation" are clearer.	We changed the language to urination throughout the manuscript.
Reviewer #2	Around line 605 you start talking about personal safety, which I have no doubt is very important to the women, but this theme is not explored much [or narrowly in the context of health] in the qualitative findings. Could you bring it out more in the data, so that when you circle back to it in the discussion you have a foundation for the claims?	In the Findings sections, we do discuss safety concerns: We first raise safety concerns on page 13, line 368. Later, on page 21, we describe the women's urination habits as "risky". Further accentuation of this point has been added on page 21. To be clear, the women's narratives did not frequently articulate safety as a concern. This is a conclusion we have raised in our analysis considering their stories about looking for dark solitary places to void outside.

Reviewer #2	I really liked your argument that the idea of carceral citizenship extends to bathroom utilization! Maybe you could expand on this some?	We further define carceral citizenship and explain how features of carceral citeznship extend to bathroom access for justice-involved women. Specifically, we draw upon findings from the focus groups to show how state and social service agencies restrict and monitor bathroom usage. In doing so, we explain how carceral citizenship affects many facets of everyday life, including the most basic need to access and use the bathroom.
Reviewer #2	A couple of articles/reports you may be interested in that focus on access to sanitation/toilets for the unhoused are listed below: Frye, Elizabeth. “Open Defecation in the United States: Perspectives from the Streets” In Environmental Justice 12 (5): 2019<br>Jessie Speer (2016) The right to infrastructure: a struggle for sanitation in Fresno, California homeless encampments, Urban Geography, 37:7, 1049-1069, DOI: 10.1080/02723638.2016.1142150<br>No Place to Go: An Audit of the Public Toilet Crisis in Skid Row.	Thank you for referring us to these resources. We read them and included them in the Introduction/Toilet Access section and Introduction/Obstacles section.
Reviewer #3	This paper focuses on the use of toilets for urination, largely missing out menstruation or defaecation. The latter are very important aspects and I was disappointed that they were not focused upon (although mentioned in the survey, and occasionally but not consistently in other places). (and admittedly would be a much wider study so my feedback is on addressing clarity of the content, not suggesting further data is needed) I think the title and introduction need to demonstrate to the reader that the focus is on use for urination – clearly the paper demonstrates the importance, but as it stands it promises a little more than it gives. The reader may assume, like myself that all aspects of toileting are focused on. Eg line 127 to 133 only talks about LUTS symptoms related to the urinary tract / bladder…… as do the interviews, please refocus the paper to reflect this narrower scope. This only needs additional phrasing here and there.	We have changed the title to clarify our focus on urination. Indeed, the instrument specifically focused on urination habits, behaviors, and experiences. We clarified the focus on urination at several points and have also changed language from "voiding" to "urination" throughout, which helps refocus the paper. The lack of inclusion about information related to other types of toilet behaviors has been included in the Limitations section.
Reviewer #3	The abstract could state where the study was carried out - in the very least the country rather than have the reader assume it from looking at where the authors were based.	Language was expanded to indicate that the city is in the US (pg 2, line 39). More information about the location is included on page 7, in the background section.
Reviewer #3	Line 251 – states how the data were organised under 3 headings?, then followed by ‘themes that surfaced ‘ appear to refer to just 2 themes ‘women’s perceptions that they could not be trusted and were being treated in ways that contested their humanity’. Were these the only themes that emerged? It might be useful to state what themes emerged if there were more. This just needs some clarity.	The use of the word "themes" in this paragraph was confusing, given the context of qualitative analysis. This terms was removed to clarify the language.

Reviewer #3	It might be useful to consider any limitations - from holding FGD for example, also considering the positionality of the researchers in terms of collecting the data and analysing it, given the specific background of the participants. Also the FGD focus on LUTS / urination behaviours but the tool used for the audit is based on a menstrual hygiene management tool - is there any mismatch? How well does it work to marry these together?	A limitations section has been added to page 27-28. This listing of limitation includes positionality of researchers. To address this reviewer's concerns about the rleationship between the audit items and the focus group instrument, further explanation about the items included in the audit has been added to the description of this instrument.
Reviewer #3	One issue that might be worth reflecting on (but not an absolute requirement) is that these are women who 'Almost all (86%) reported a history of substance use, with 26% reporting injection drug use'. In their narratives participants talked about many private facilities are kept locked for customer use to prevent the facilities being used for drug taking. Did the women discuss this dichotomy? Were they questioned about whether they use or had used toilets as a place to take drugs, or if they understood why facilities might be kept closed for this reason and if they could suggest a solution. I think this is a real dilemma and can see how important it is for public facilities to be available to all, but understand why they might not be. Do the authors have any solutions - this is a problem we found in our own menstrual study, so not just US based.	We did not ask participants if they had used public or private bathroom facilities to use drugs. A paragraph (pp. 34) has been added to the discussion section to address these issues.
Reviewer #3	Can you check the research evidence on line 34 ' Indeed, squatting to urinate on a consistent basis can weaken the bladder muscles and result in lower urinary tract symptomology (LUTS) or pelvic organ prolapse (33, 45]. The reference states it 'may' suggesting not conclusive, also there is research suggesting the alternative, that a squatting technique as usual in many Asian countries for example can strengthen the pelvic floor. It may be that the authors clarify that squatting / hovering over a toilet may be unhealthy.	Language was modified to indicate that squatting over a toilet may be unhealthy. (p. 31, line 869)
Reviewer #3	The line 640 'Research has demonstrated that fines associated with "quality of life" charges can lead to incarceration and homelessness for those who are unable to pay [50]. As a reader I an not quite sure what a quality of life charge means, particularly in relation to outside urination - can this be clarified please?	We further explain that quality-of-life charges and citations are minor offenses, such as public urination or loitering, that often target the poor. We provide an example from the literature that demonstrates how the inability to pay the fines and fees associated with minor, quality-of-life charges leads to warrants, arrest, and further worsens poverty for society's most disenfranchised.

Reviewer #4	Line 30: Bathroom(s) is a word used in the US only. If targeting a broader audience, the paper needs to define the words: bathroom, restroom, and toilet. Throughout the paper, authors interchangeably use "toilets," "restroom," and "bathroom" which they do not mean the same thing. I would recommend authors to first, be consistent with the words used throughout the paper. If the focus is on toilet access, then define it based on the WHO/UNICEF Joint Monitoring Programme (JMP) definition of securing basic access to sanitation. But do not go back and forth without really defining what a bathroom or restroom mean throughout the paper as these words do not mean the same thing and are not used worldwide. For WaSH scholars, the word used is typically sanitation, which refers to toilets. Would recommend doing more reading on the definitions to include in the paper. There is some literature that states bathroom refers to the private home vs. restroom refers to public facilities with a toilet and a sink to practice hand hygiene. It is important to distinguish and clearly define each of these terms. https://washdata.org/monitoring.	Definition of "restroom" were added to the beginning of the manuscript (p. 3, lines 69-71) and the paper was edited to use this term consistently.
Reviewer #4	Line 119: remove bathroom, it should be toilet access. Be consistent and revise throughout the paper based on definitions.	Language changed from bathroom to toilet access.
Reviewer #4	Lines 169-180: Consider moving to the results section. It does not belong in the methods section as this is a finding (pop demographics). Similar with lines 230-236, starting with "All the bathroom were located..." And ending with "private institutions (n-11, 55%)," could be moved to the results when referring to audit of toilets. This is a result and does not belong in methods section either.	Both of these sections were moved from Methods to Results. The participant demographics were moved to page 13. The section that had started with "All the bathrooms were located..." was moved to p. 25.
Reviewer #4	Line 178: Define "ever being homeless." Does this mean that if at any time in their lives these women have experienced homelessness? In the past 6-months? Or are they currently experiencing homelessness?	This sentence was edited for clarity. This statistics relates to being homeless at any point in their lives. We did not ask specifically about last 6 months or current situation.
Reviewer #4	Line 178-179: Not clear from this pop characteristics, the number of women who are unstably housed or currently experiencing homelessness and need to specify in the results section. It would also be helpful to state the number of women who are housed (either living with family, with friends), shelter systems, and or who were unhoused at the time study activities were conducted. It would also be helpful to include who is employed and unemployed (aside from describing the work experiences), to provide a bit more information on population characteristics to understand the population without identifying personal information about these study participants. This would also help contextualize women's different access to sanitation based on place of employment described in section located in 318.	We did not collect this information. We did not ask participants where they were currently living or working.

Reviewer #4	Lines 287-291: Highlights an important argument and would like to see if the authors could expand on this point about discriminatory practices. What about the Black, Latina/Latine, and other underrepresented racial/ethnic women in the sample, did they highlighted any experience any of discrimination when trying to access a toilet in public spaces? Incorporate an intersectional analysis. What about women in different age groups, which this is slightly highlighted in lines 497 - 505. Highlight what are the intersectional vulnerable identities among these justice-involved women?	We cannot do this type of racial or ethic analysis because our data does not identify the race, ethnicity or age of any specific speaker. The demographic data relects the demographics of the focus group, but not the individuals in the focus group. This methodological issue was added to the Limitations section.
Reviewer #4	Are there any women who menstruate in the sample population and do these women encounter challenges in menstrual hygiene management with limited access to a toilet? This is helpful to know given that on line 552 there is a mention on audit of toilets that rarely accommodate people who menstruate.	We did not ask participants if they menstruate nor did we ask question about MHM.
Reviewer #4	Line 640: "Quality of life" charges, do you mean sleeping outdoors? Loitering? Would be helpful to define and highlight what city ordinances exist in the town where this study takes place and that perpetuates a cycle of poverty.	We further explain that quality-of-life charges and citations are minor offenses, such as public urination or loitering, that often target the poor. We provide an example from the literature that demonstrates how the inability to pay the fines and fees associated with minor, quality-of-life charges leads to warrants, arrest, and further worsens poverty for society's most disenfranchised. We did not, however, add any of the ordinances from the location of our study, since we have chosen to not disclose the location of the study.
Reviewer #4	Line 545: Recommend authors to highlight what is consider an excellent (point 5) facility. What do these facilities/toilets need to have been ranked a 1 vs 5-point? Helpful to describe in the methods section. Authors mentioned this is adapted from Maroko et al. (2021) article but should consider including a description of this point classification in the methods.	Explanation of the point system was added to the Methods section, p. 11-12, lines 319-330
Reviewer #4	Line 518: Is there publicly data available on the total number of public toilet facilities available in the town where this study takes place? To understand how many were not audited/missed. Authors could consider looking into this but not necessary, to highlight that only a portion of facilities were audited and not all.	No. There is no publicly available data about the number of public toilet facilities in the city.

Reviewer #4	Consider reading "No-Place to Go" audit report on toilet access in Skid Row, Los Angeles, CA and the Dirty Divide: Out of Service report by LACAN in Skid Row, Los Angeles, CA to connect existing studies in the discussion with the study contributions. At the same, consider engaging debates about urban space, homelessness, criminalization, and public service provision by including the work of Eugene McCann, Jessie Speer, Erin Goodling, Jennifer Wolsch, Don Mitchell, Forrest Stuart, Kelly Lytle Hernandez, Alheli Calderon-Villarreal, Lourdes Johanna Avelar Portillo, Shawn Flanigan, and Megan Welsh. The work of these authors provides helpful insights and discussions that are absent in this manuscript and can make the discussion stronger.	Thank you for bringing this scholarship on homelessness, drug use, and social control to our attention. We recognize the link between returning from prison, homelessness, and social control. However, our focus was specifically on justice-involved women and aimed to underscore that lack of toilets is a reentry issue and a challenge for all people, unhoused or not. We have references "no place to go", Speer (2016); Frye et al (2019); and Amato et al (2022) to the Introduction and Discussion sections.
Reviewer #4	Line 40: I would clearly define the word "justice involved women" could write, justice involved women refers to women who...as not everyone may know the term and helpful for broader audience.	This sentence has been edited to clarify the definition of this term is being provided. (p. 3).
Reviewer #4	I think it would be important to contextualize the location of this small town. If the issue of not digging deeper is to maintain confidentiality, then I would understand, but it is always important to understand the location of where this study is based on. Is this a rural town, how many people in this town and how many of those are unhoused/people experiencing homelessness? Important to know what the number of justice-involved women in the town is particularly when trying to fill knowledge gaps. This should go somewhere in the background section before line 81. Authors briefly mention this in line 584 but it takes many pages to learn a bit about the town.	The location of this study is already described as a "small urban area in the northeastern region of the United States". We have added information about the population of the town, the number of unhoused people, and reentry population (p. 7-8).
Reviewer #4	Line 59-60: Authors mention that "Black and Latina women are disproportionately incarcerated, and the majority of incarcerated women are white." Based on your study findings, it would be important to highlight this in the results section. Are there any differences in access and experiences of discrimination among the women who participated in the focus groups in their access to public/private toilets? This would make the analysis stronger. Incorporating an **intersectionality** analytical lens could also help make the results and discussion stronger (see major comment R1C5).	As described in previous item, we do not have the data to do this type of intersectional analysis. We have noted this limitation in the Limitations section.
Reviewer #4	Line 121-131: Could highlight better literature on WaSH insecurity, particularly literature on psychosocial distress related to limited access to sanitation and hygiene services. There are existing studies that explore the intersections of sanitation and hygiene insecurity and psychosocial stress among women.	Thank you for bringing our attention to the WaSH literature. Given this article's focus on the US, we have decided not to include this research which primarily focuses on non-US locations.

Reviewer #4	Consider citing article on Kuhlmann et al. (2019) on menstrual hygiene for low-income women in the US that may be useful to include in both literature/background section and discussion.	Reference to the Kuhlmann article was added to the Introduction/Toilet Access section (p. 5, lines 121-123).
Reviewer #4	Line 30: remove "to" in the sentence "limited to access" as it should read limited access to _____.	This "to" has been removed.
